# Simultaneous Transfer of Leaf Rust and Powdery Mildew Resistance Genes from Hexaploid Triticale Cultivar Sorento into Bread Wheat

**DOI:** 10.3389/fpls.2018.00085

**Published:** 2018-02-05

**Authors:** Feng Li, Yinghui Li, Lirong Cao, Peiyuan Liu, Miaomiao Geng, Qiang Zhang, Lina Qiu, Qixin Sun, Chaojie Xie

**Affiliations:** Key Laboratory of Crop Heterosis and Utilization, Ministry of Education, State Key Laboratory of Agro-Biotechnology, Beijing Key Laboratory of Crop Genetic Improvement, China Agricultural University, Beijing, China

**Keywords:** hexaploid triticale, powdery mildew, leaf rust, resistance, marker analysis, GISH, FISH

## Abstract

Wheat powdery mildew, caused by *Blumeria graminis* f. sp. *tritici*, and wheat leaf rust, caused by *Puccinia triticina* Eriks, are two important diseases that severely threaten wheat production. Sorento, a hexaploid triticale cultivar from Poland, shows high resistance to the wheat powdery mildew isolate E09 and the leaf rust isolate PHT in Beijing, China. To introduce resistance genes into common wheat, Sorento was crossed with wheat line Xuezao, which is susceptible to both diseases, and the F_1_ hybrids were then backcrossed with Xuezao as the recurrent male parent. By marker analysis, we demonstrate that the long arm of the 2R (2RL) chromosome confers resistance to both the leaf rust and powdery mildew isolates at adult-plant and seedling stages, while the long arm of 4R (4RL) confers resistance only to powdery mildew at both stages. The chromosomal composition of BC_2_F_3_ plants containing 2R or 2RL and 4R or 4RL in the form of substitution and translocation were confirmed by GISH (genomic *in situ* hybridization) and FISH (fluorescence *in situ* hybridization). Monosomic and disomic substitutions of a wheat chromosome with chromosome 2R or 4R, as well as one 4RS-4DL/4DS-4RL reciprocal translocation homozigote and one 2RL-1DL translocation hemizigote, were recovered. Such germplasms are of great value in wheat improvement.

## Introduction

Wheat, accounting for 25% of total global cereal yield, provides 20% of the calories consumed by humans (FAO, 2014). Breeding wheat with high yield and good quality has been regarded as a pivotal component of efforts to satisfy food demand of the world. However, wheat diseases such as powdery mildew and leaf rust severely threaten the production of common wheat. Since chemical control always brings environmental pollution and health threats, the use of host resistance is considered an environmentally benign and effective way to control these diseases (Murray and Brennan, 2009).

Wheat powdery mildew (PM), caused by the obligate biotrophic fungal pathogen *Blumeria graminis* f. sp. *tritici* (*Bgt*), is a destructive disease of common wheat in areas with cool or maritime climates. The disease can lead to severe yield losses ranging from 13–34% (Griffey et al., 1993; Conner et al., 2003). Another fungus, *Puccinia triticina* Eriks (*Pt*), the causal agent of wheat leaf rust (LR), drastically decreases wheat yield across the world and has afflicted wheat for thousands of years (Bolton et al., 2008; Huerta-Espino et al., 2011). Hitherto, approximately 57 *Pm* (powdery mildew) and 76 *Lr* (leaf rust) resistance loci have been designated (McIntosh et al., 2014; Hao et al., 2015; Ma et al., 2015; Zhang et al., 2016; Bansal et al., 2017; Liu et al., 2017; Singla et al., 2017). Most of them are race-specific and only a few were characterized as durable resistance such as *Lr34, Lr46*, and *Lr67* (Krattinger et al., 2009; Ellis et al., 2014; Moore et al., 2015). Race-specific resistance genes tend to be overcome within several decades due to rapid evolution of physiological races and strong selection for virulent pathogen mutants (Niewoehner and Leath, 1998; Parks et al., 2008; Huerta-Espino et al., 2011; Zeng et al., 2014). Therefore, continuously exploiting naturally occurring resistance genes remains the most effective measure to improve the disease resistance of bread wheat.

Rye (*Secale cereale* L.), a relative of wheat, has been utilized intensively in wheat breeding programs due to its excellent stress tolerance and disease resistance (Jiang et al., 1994; Friebe et al., 1996; Rabinovich, 1998; Purnhauser et al., 2010). The short arm of the rye 1R chromosome carries abundant resistance genes, such as *Pm8, Pm17, Sr31, Sr50, Yr9*, and *Lr26*, and has been introduced to wheat in the form of T1BL.1RS and T1AL.1RS (Mcintosh et al., 1988; Heun and Friebe, 1990; Singh et al., 1990; Mago et al., 2004, 2015; Hurni et al., 2013). Wheat varieties containing this fragment always display high yield potential, broad adaptation and disease resistance against powdery mildew, stem rust, leaf rust, and stripe rust (Villareal et al., 1995, 1998; Ehdaie et al., 2003; Kim et al., 2004), however, the *Sec-1* locus carried on 1RS is detrimental to bread-making quality when it replaces the *Glu-3* and *Gli-1* genes of wheat (Dhaliwal et al., 1987; Dhaliwal and MacRitchie, 1990; Martin and Stewart, 1990). Besides 1RS, rye chromosomes 2R, 4R, and 6R also possess resistance genes against powdery mildew and leaf rust. *Pm20* on chromosome arm 6RL from rye cv. Prolific was translocated onto wheat chromosome 6BS (Friebe et al., 1994). *Pm7* was located on 2RL and is present in the form of T4BS.4BL-2RL in the wheat germplasm Transec (Friebe et al., 1996). The long arm of rye chromosome 2R has been reported to carry resistance genes against wheat powdery mildew and leaf rust diseases, and the long arm of chromosome 4R from rye cv. Kustro were also reported to confer resistance to wheat powdery mildew (Friebe et al., 1996; An et al., 2006, 2013; Hysing et al., 2007; Zhuang et al., 2010; Fu et al., 2014). However, due to the coevolution of host and pathogen, the resistance genes (*Pm8, Pm17, Sr31, Yr9*, and *Lr26*) on 1RS and *Pm7* on 2RL are no longer effective in China and some other parts of the world, and virulence against *Pm20* has arisen (Lutz et al., 1992; Zhuang and Li, 1993; Niewoehner and Leath, 1998; Imani et al., 2002; Zhuang, 2003; Zeng et al., 2014; Hubbard et al., 2016).

Hexaploid triticale (× *Triticosecale* Wittmack, AABBRR, 2*n* = 6*x* = 42), synthesized artificially by combining the genomes of *Triticum turgidum* (AABB, 2*n* = 4*x* = 28) and *S. cereale* (RR, 2*n* = 2*x* = 14), possesses outstanding resistance to wheat powdery mildew and leaf rust disease (Oettler, 2005). The rye components in triticale have been adapted to the wheat nucleus and cytoplasm, which renders easier the transfer of rye chromosomes into common wheat (Ma and Gustafson, 2008). By contrast, colchicine treatment and tedious embryo rescue are indispensable for making wheat × rye crosses (Oettler, 1983, 2005). Thus, triticale cultivars can serve as an alternative source in wheat improvement. The R- and D-genome chromosomes in progenies of triticale × wheat crosses were mostly present as univalent in triticale × wheat F_1_ hybrids (AABBDR) during meiosis (Schlegel et al., 1980; Lukaszewski and Gustafson, 1983). Univalents tend to misdivide at anaphase I followed by the fusion of telecentric chromosomes during interkinesis of meiosis II which results in whole-arm Robertsonian translocations (Friebe et al., 2005). In this way, wheat-rye translocations can be produced within homoeologous or non-homoeologous groups in sufficient numbers (Lukaszewski and Gustafson, 1983). The rye chromosomes in triticale × wheat F_1_ hybrids can be transmitted through egg cells at a higher frequency than through pollen, and the transmission of individual rye chromosomes in F_1_ × wheat was around 50% except for 6R, which shows a significant lower frequency than random (Lukaszewski et al., 1982). Triticale cultivar originating from hybridization of several triticale lines varied in the rye genomes represents the diversity of rye. It combines the broad stress tolerance of different triticale lines and can be used to improve the powdery mildew and rust resistance of wheat. The direct application of triticale cultivar × wheat crosses can facilitate the exploration of multiple resistance genes simultaneously in a short time.

Molecular markers facilitate the identification of alien chromosome segments and have been extensively adopted in breeding programs (Shimizu et al., 1997; Song et al., 2008). To date, a large number of PCR-based markers, such as EST, SSR, and EST-SSR markers for marker associated selection (MAS), have been developed and mapped to specific rye chromosomes, and most of them have been employed in the identification of target rye chromosomes (Saal and Wricke, 1999; Hackauf and Wehling, 2002; Khlestkina et al., 2004; Lee et al., 2009; Xu et al., 2012; Martis et al., 2013; Nguyen et al., 2015). These markers enable breeders to identify the specific segments of rye chromosomes present in wheat germplasm at a large scale. Cytological methods such as GISH (genomic *in situ* hybridization) and FISH (fluorescence *in situ* hybridization) have been used to detect the rye chromosomal fragments in wheat-rye hybrids (Tsuchida et al., 2008; Zhuang et al., 2010; Fu et al., 2014). *S. cereale* clone pSc119.2 mainly hybridizes to B-genome chromosomes of wheat and R-genome of rye, and *Aegilops squarrosa* clone pAs1 produces signals especially on D-genome chromosomes. Multicolor fluorescence *in situ* hybridization (mc-FISH) using these probes allows the identification of most wheat chromosomes and all rye chromosomes (Schneider et al., 2003; Contento et al., 2005; Tang et al., 2014). By combining molecular marker screening and cytological analysis, chromosomal organization in terms of translocations, substitutions and additions can be characterized in triticale-wheat derivatives easily.

Triticale cultivar Sorento is highly resistant to wheat powdery mildew and leaf rust diseases as well as triticale diseases. However, the resistance has not been studied and exploited in wheat breeding. In the present study, to incorporate resistance genes against powdery mildew and leaf rust into bread wheat, Xuezao, a wheat line susceptible to powdery mildew and leaf rust, was crossed with the resistant triticale cv. Sorento as the female parent. The F_1_ hybrids were then backcrossed with Xuezao as the recurrent male parent for several rounds. By means of marker analysis and cytological analysis, it is possible to locate resistance genes on rye chromosomal arms in a wheat background. Here, we report the identification of powdery mildew and leaf rust resistances, both at adult and seedling stage, conferred by chromosome arms 2RL and 4RL of Sorento. Several lines stable genetically were produced, which represent valuable germplasms that can be used in wheat improvement.

## Materials and methods

### Materials

**Figure 1:**
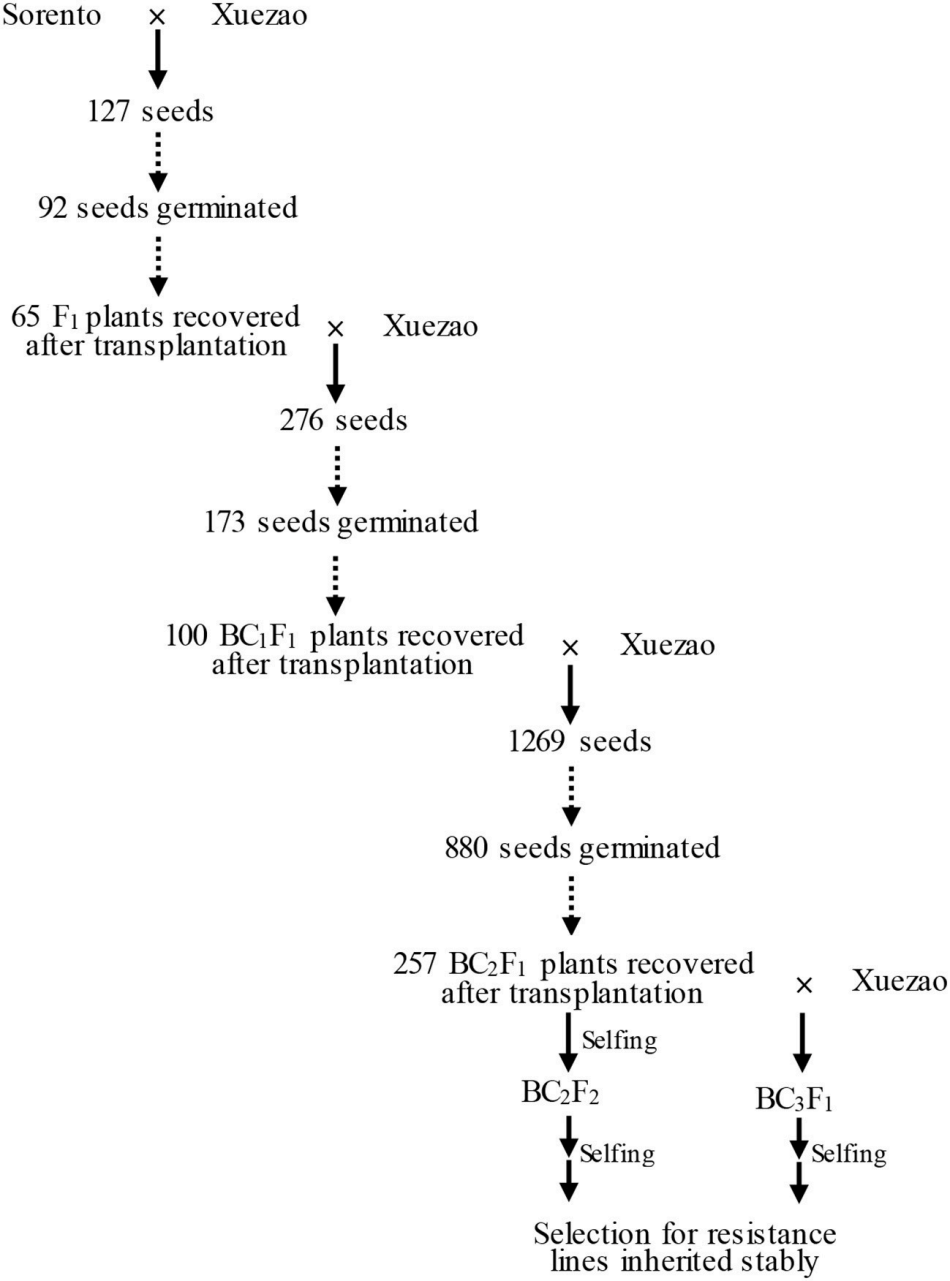
Procedure for transferring resistance from Sorento into Xuezao.

Hexaploid triticale cv. Sorento, highly resistant to wheat powdery mildew and leaf rust disease strains present in Beijing, China, was kindly provided by Danko Hodowla Roślin Sp. z o.o., Choryn, Poland (http://danko.pl/odmiany/sorento/?lang=en). Sorento is a winter triticale cultivar from Poland that yields very well and is remarkably resistant to most triticale diseases. Triticale cv. Sorento derives from a three-way cross of different triticale lines; its pedigree is CT932-89/CHD610-86//Moreno. All those lines in their deep pedigree are based on lines from CIMMYT, Canada and Hungary. The wheat line Xuezao is a winter wheat susceptible to both diseases in Beijing. Triticale cv. Sorento and bread wheat Xuezao were planted in the field at Shang Zhuang Experimental Station in the autumn of 2012. To introduce resistance from Sorento into Xuezao, we designed a backcrossing strategy in which Sorento acts as the donor parent and Xuezao as the recurrent parent (Figure 1). Each autumn during the experiment, both parents and their progenies were evaluated for powdery mildew resistance at seedling stage in the greenhouse at China Agricultural University. The surviving plants were transplanted into the field at Shang Zhuang Experimental Station in Beijing to evaluate their powdery mildew and leaf rust disease symptoms at adult-plant stage. Under our condition, Sorento initiates flowering about 15 days later than Xuezao. In order to minimize the time gap, Sorento and the progenies were covered with plastic film in which a relative high temperature can be maintained. Such condition allows plants to grow faster in spring as well as avoid winterkill.

### Field crosses

Sorento was crossed with Xuezao as the female parent in May in 2013. All the recovered Sorento × Xuezao F_1_ plants (1–5 spikes per plant) were backcrossed. One to seven spikes of BC_1_F_1_ and BC_2_F_1_ plants showing resistance to powdery mildew or leaf rust diseases were pollinated with pollen of Xuezao. Field crosses were carried out each May from 2012 to 2016.

### Evaluation of powdery mildew symptoms in field and greenhouse

The phenotypes of both parents and BC_1_F_1_, BC_2_F_1_, BC_3_F_1_, and BC_2_F_2_ progenies were evaluated at the adult-plant stage in the field and at the seedling stage in the greenhouse. For seedling tests, both parents and their progenies were sown into 50-pot trays containing three parts nutrient soil and one part vermiculite. Wheat powdery mildew isolate E09, provided by the Institute of Plant Protection of the Chinese Academy of Agricultural Sciences in Beijing, was maintained and multiplied on susceptible line Xuezao. After about a week, the unfolded first leaf was inoculated with powdery mildew by hand flick. The infection types (IT) of 10–15 days post-inoculation (dpi) plants were scored using the 0–4 scale described by Zhang et al. (2010), in which “0,” “0;”, “1,” “2,” “3,” and “4” indicate “no visible symptoms,” “necrotic flecks without sporulation,” “highly resistant,” “resistant,” “susceptible,” and “highly susceptible,” respectively. All plants were kept under a day/night photoperiod of 18/6 h at 25–30°C in the greenhouse. For field tests, wheat seedlings with well-developed powdery mildew hyphae were used as media for spore transmission and inoculation to the overwintered wheat after being transplanted into the field in April. After 1–2 months, the infection types of the flag leaf and the top second leaf of the progeny were evaluated using the same standard.

### Evaluation of leaf rust symptoms in field and growth chamber

Evaluation of leaf rust was carried out in BC_1_F_1_, BC_2_F_1_, BC_3_F_1_, and BC_2_F_2_ in the field and in BC_2_F_2_ in growth chamber using leaf rust isolate PHT provided by the Institute of Plant Protection of the Chinese Academy of Agricultural Sciences in Beijing. For field tests, each progeny plant that overwintered was inoculated with leaf rust spores by injecting the urediniospore solution containing 0.1% Tween 20 into the stalk in April. After 1–2 months, the infection type of the flag leaf and the top second leaf of each individual was scored on a 0–4 scale, with “0” for immune (no uredinia), “;” for nearly immune (no uredinia, but hypersensitive necrotic or chlorotic flecks present), “1” for very resistant (small uredinia surrounded by necrosis), “2” for moderately resistant (small to medium-sized uredinia often surrounded by chlorosis or necrosis), “3” for moderately susceptible (medium-sized uredinia that may be associated with chlorosis), and “4” for susceptible (large uredinia without chlorosis) (Roelfs et al., 1992). Seedling tests were only performed on self-crossed progenies (BC_2_F_2_) of BC_2_F_1_ plants showing resistance to leaf rust at the adult-plant stage in the plant growth chamber. Seven- to eight-day-old plants were challenged with leaf rust by spraying urediniospore solution. The inoculated plants were then incubated in a humid growth chamber free from light for 1–2 days. After inoculation, the plants were maintained under a day/night photoperiod of 18/6 h, a temperature of 25–30°C, and a high relative humidity. Xuezao was taken as the susceptible control. After around 10 days, the infection type was scored as described for adult plants.

### Markers for identification of rye chromosomes and resistance loci

A total of 64 published chromosome-specific rye markers, including REMS (rye expressed microsatellite sites), SCM (*Secale cereale* microsatellite), GRM, SWES, and a few other types, were chosen to identify the rye chromosomes 1R-7R of triticale (Saal and Wricke, 1999; Hackauf and Wehling, 2002; Khlestkina et al., 2004; Zhuang et al., 2010; Xu et al., 2012; Martis et al., 2013; Nguyen et al., 2015). The number of markers on each rye chromosome ranges from 4 to 25 with an average of 9. All rye markers and their features are listed in the Table S1. All markers were screened for polymorphisms between Xuezao and Sorento by PAGE (polyacrylamide gel electrophoresis) analysis, and the polymorphic markers were then used to genotype the backcrossed individuals.

### Molecular marker analysis

In the present study, PCR-based identification was used to detect the alien chromatin in wheat-triticale derivatives. Total leaf genomic DNA of Sorento, Xuezao, and their progenies was isolated using the CTAB method with some modifications (Allen et al., 2006). PCR amplifications were conducted in a 10 μL mixture containing 10 mM Tris-HCl, 50 mM KCl, 1.5 mM MgCl_2_, 200 μM dNTPs, 0.75 U Taq DNA polymerase, 0.2 μM of each primer, and 50–100 ng genomic DNA. The PCR program consisted of an initial denaturation at 94°C for 5 min followed by 40 cycles of 94°C for 45 s, 50–60°C for 35 s, and 72°C for 35 s, followed by a final extension at 72°C for 10 min. The PCR products were analyzed on 8% non-denaturing polyacrylamide gels, and the gels were then silver stained and photographed.

### Cytological identification of rye-derived chromosomes

Cytological analysis was carried out using GISH and FISH as described by Han et al. (2006). Triticale-wheat substitution, translocation or addition lines resistant to wheat powdery mildew or leaf rust in BC_2_F_3_ were selected for cytological analysis. For GISH analysis, the rye genome was used as a probe to detect the rye-derived chromosomal segments in Sorento. For FISH analysis, the probes pSc119.2 and pAs1, containing highly repetitive sequence of wheat and rye, were used to distinguish the wheat A-, B-, and D-genomes and the rye R-genome. Probes pSc119.2 and pAs1 were labeled with Fluor-488-5-dUTP (Invitrogen) and Texas red-5-dCTP (Invitrogen), respectively as described by Wang et al. (2017). Chromosome preparation and *in situ* hybridization were carried out according to Han et al. (2006). Images were taken with epifluorescence microscope Olympus BX61.

## Results

### Introgression of the powdery mildew and leaf rust resistance by backcrossing

To introduce disease resistance from triticale to wheat, the resistant triticale cultivar Sorento was crossed with the susceptible wheat line Xuezao to create the F_1_ hybrids. The seed setting rate in the cross Sorento × Xuezao was 7.1%. Among 127 seeds sown, 92 (72.4%) germinated. Only 65 (70.6%) of such plants survived after inoculation and transplantation into field (Figure 1). All F_1_ plants were completely male sterile, as revealed by open glumes and aborted anthers after heading. The rate of seed setting of F_1_ × Xuezao backcross ranged from 0 to 20% and a total of 276 seeds were obtained with the total germination and recovery rate of 62.7 and 57.8%, respectively (Figure 1). Nearly 90% of the resulting BC_1_F_1_ plants remained male sterile. The total germination rate in BC_2_F_1_ was 69.3%, whereas the recovery rate was only 29.2% because the plastic film got damaged and most of the transplanted plants were winterkilled (Figure 1). However, the average fertility improved in the BC_2_F_1_ and BC_3_F_1_ plants.

All geminated and recovered F_1_ plants showed immunity to both powdery mildew isolate E09 and leaf rust isolate PHT at the adult and seedling stage (Table 1). Of 173 BC_1_F_1_ individuals challenged with powdery mildew in the greenhouse, 108 plants showed seedling resistance. This indicates that the transmission rate of the powdery mildew resistance at seedling stage through egg cells of Sorento × Xuezao F_1_ hybrids was 62.4% (Table 1). At adult-plant stage, the occurrence of both powdery mildew and leaf rust was investigated in 100 BC_1_F_1_ plants. A total of 49 and 63 adult plants were resistant to leaf rust and powdery mildew respectively, though 73 plants did not survive after transplantation (Table 1). We found that all plants resistant to leaf rust also displayed excellent resistance to wheat powdery mildew in BC_1_F_1_ and BC_2_F_1_. For plants susceptible to leaf rust, the degree of the development of powdery mildew uredospores varied.

**Table 1:** Inheritance of powdery mildew and leaf rust resistance in progeny.

	**F**_**1**_		**BC**_**1**_**F**_**1**_			**BC**_**2**_**F**_**1**_		
	**No. of resistant plants**	**Total no. of plants evaluated**	**No. of resistant plants**	**Total no. of plants evaluated**	**Transmission rates (%)**	**No. of resistant plants**	**No. of susceptible plants**	**Total no. of plants evaluated**
PM at seedling stage	117	117	108	173	62.43	367	513	880
LR at adult-plant stage	65	65	49	100	49.00	152	105	257
PM at adult-plant stage	65	65	63^a^	100	63.00	194^a^	63	257

a *The plants resistant to leaf rust were also incompatible with powdery mildew and for plants susceptible to leaf rust, the degree of the development of powdery mildew uredospores varied. Thus, the number of plants resistant to powdery mildew at the adult-plant stage may not be precise*.

### Marker analysis of powdery mildew resistance at seedling stage

To select rye markers for use in our experiments, 64 markers were first screened for polymorphism between Sorento and Xuezao (Table S1). A total of 56 markers were polymorphic and 51 could be used to unambiguously identify the rye chromosomes present in the BC_1_F_1_ backcrossed progenies. The transmissions of different rye chromosomes of Sorento were first investigated on 64 BC_1_F_1_ germinated seedlings using these specific markers. The number of the rye chromosomes transmitted through F_1_ egg cells ranged from 0 to 6 with an average number of 3.09. Most of rye chromosomes showed a transmission rate of about 50% except for 3R and 6R, which showed a much lower frequency (Table 2). The average transmission rate was 44.2%.

**Table 2:** Transmission rate of individual rye chromosome in F_1_ and BC_1_F_1_ through female gametes.

**Combination**	**No. of plants analyzed**	**No. of rye chromosomes**						
		**1R**	**2R**	**3R**	**4R**	**5R**	**6R**	**7R**
Sorento × Xuezao	65	65 (100%)	65 (100%)	65 (100%)	65 (100%)	65 (100%)	65 (100%)	65 (100%)
F_1_ × Xuezao	64	33 (51.6%)	33 (51.6%)	16 (25.0%)	35 (54.7%)	29 (45.3%)	21 (32.8%)	31 (48.4%)

To locate the powdery mildew resistance of Sorento at seedling stage, the polymorphic rye markers on chromosomes 1R-7R were used to analyze the chromosome composition of 284 BC_2_F_1_ individuals derived from 9 BC_1_F_1_ plants with different rye constitutions (Table S1). The result showed that 125 plants resistant to powdery mildew isolate E09 carried either chromosome 4R or 2R, or both. Specifically, 57 plants carrying the 4R chromosome showed immunity (IT = 0) or high resistance (IT = 0; or 1) to wheat powdery mildew and 33 plants carrying the 2R chromosome only showed moderate resistance (IT = 2) at 10 dpi (Figures S1A,C, **Table 4**). The remaining 35 BC_2_F_1_ plants were immune or highly resistant to powdery mildew and contained both chromosomes 4R and 2R (**Table 4**). Plants lacking 4R and 2R were highly susceptible (IT = 4) (Figures S1A,C, **Table 4**). At 16–18 dpi, in contrast to the plants carrying 4R (IT = 0, 0; or 1), all plants only carrying 2R were susceptible (IT = 3). This demonstrated that in these BC_2_F_1_ plants, the 2R chromosome from Sorento slowed down the infection progress at the seedling stage.

**Figure 2:**
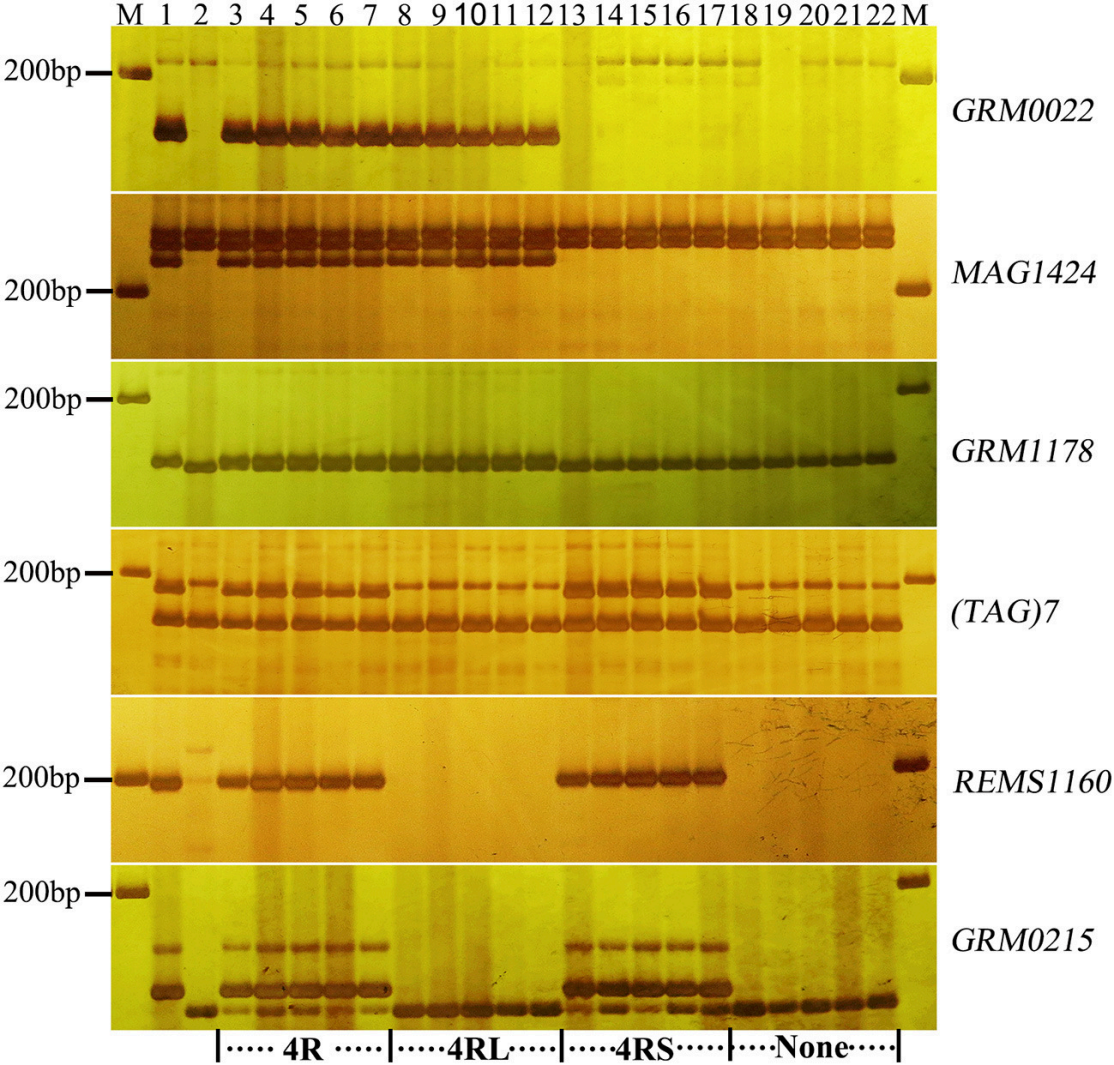
Marker analysis of BC_2_F_2_ plants segregating for 4R using rye-specific markers (*GRM0022, MAG1424*, and *GRM1178*) on the long arm of 4R chromosome and markers (*(TAG)7, REMS1160*, and *GRM0215*) on the short arm of 4R chromosome. Ten plants (lane 3–12) resistant to powdery mildew at the seedling stage and 10 susceptible plants (lane 13–22) were analyzed. Lane 3–7 and 18–22 show plants with entire 4R and without 4R chromosome, respectively. Lane 8–12 and 13–17 signify the presence of 4RL and 4RS alone, respectively. Lane 1 and lane 2 signify the resistant parent (Sorento) and susceptible parent (Xuezao), respectively.

**Figure 3:**
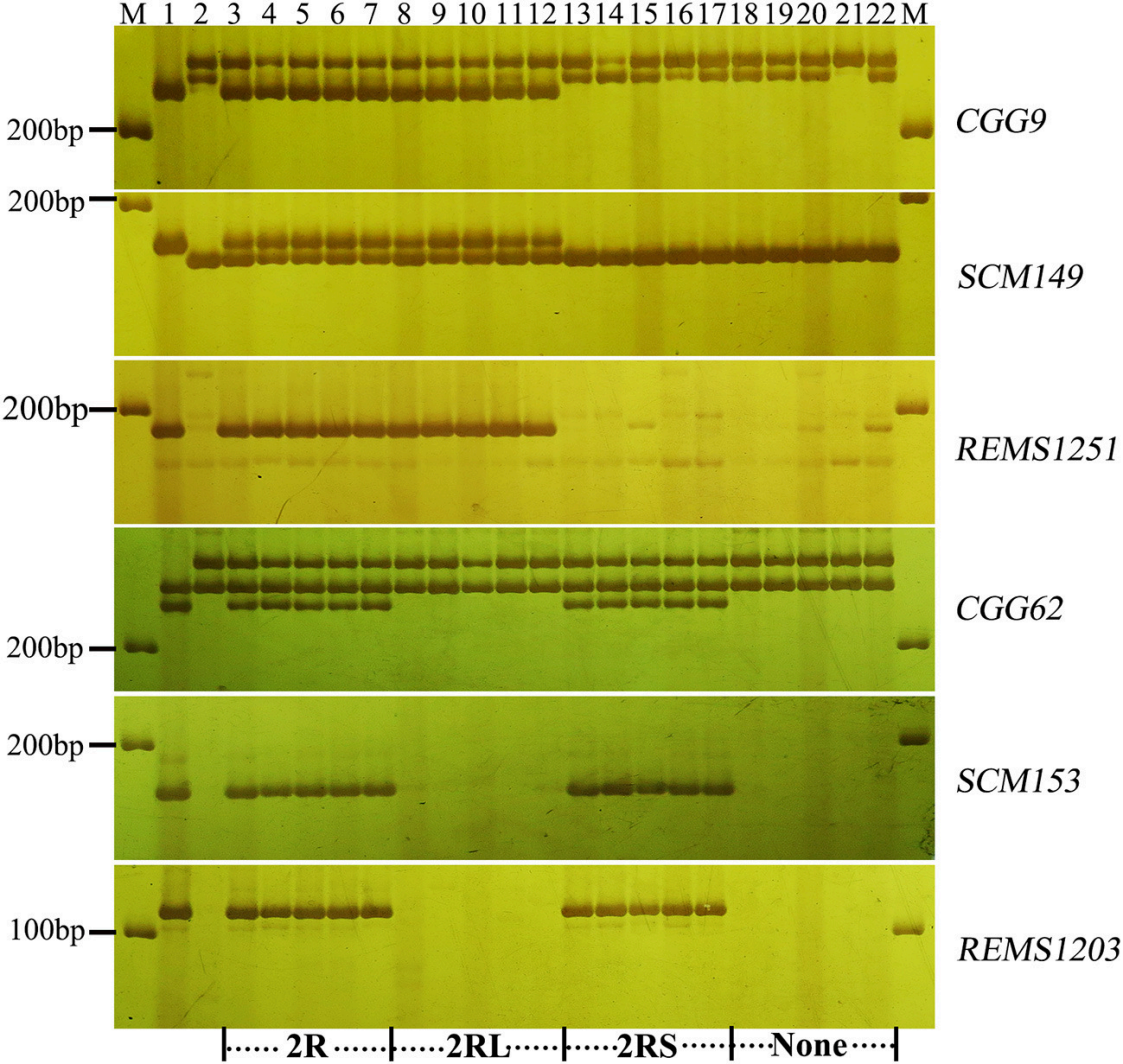
Marker analysis of BC_2_F_2_ plants segregating for 2R using rye-specific markers (*CGG9, SCM149*, and *REMS1251*) on the long arm of 2R chromosome and markers (*CGG62, SCM153*, and *REMS1203*) on the short arm of 2R chromosome. Ten plants (lane 3–12) resistant to powdery mildew at the seedling stage and 10 susceptible plants (lane 13–22) were analyzed. Lane 3–7 and 18–22 show plants with entire 2R and without 2R chromosome, respectively. Lane 8–12 and 13–17 signify the presence of 2RL and 2RS alone, respectively. Lane 1 and lane 2 signify the resistant parent (Sorento) and susceptible parent (Xuezao), respectively.

To validate the resistance conferred by 4R and 2R from Sorento at the seedling stage, 234 and 181 BC_2_F_2_plants segregating only for 4R and 2R, respectively, were surveyed using chromosomal arm-specific markers (Table 3). In the 4R experiment, a total of 106 resistant individuals were found to carry the 4R chromosome, among which 21 plants contained only the long arm of 4R. The remaining 128 susceptible plants did not contain 4RL, but 16 of these plants contained 4RS (Figure 2). This further suggests that 4RL from Sorento confers excellent resistance to wheat powdery mildew. In the 2R experiment, 70 plants were characterized as resistant to powdery mildew; the resistance was located on the long arm of 2R chromosome, as proven by markers from 2RL (Figure 3). However, plants carrying any of the other five rye chromosomes of Sorento were shown to be completely susceptible to wheat powdery mildew at the seedling stage.

**Table 3:** Arm-specific markers used for detecting rye chromosomes 2R and 4R.

**Chromosomes**	**Marker**	**Forward sequence**	**Reverse sequence**	**Type**	**SSR**	**Expected size(bp)**	**References**
2RS	*CGG62*	GCCCTCGACGACATGAAA	CGCTTGCCGGTCTTGTAT	EST		290	Xu et al., 2012
2RS	*SCM153*	CACTATGATAACCGTACCTCAA	AATACTGCACGTAGGAATCAAC	EST-SSR	(AT)9	170	Hackauf and Wehling, 2002
2RS	*GRM1082*	TCTAGCTCATCAAGTGCTTACCA	GTTCCGCTGAGATGAACCATA	EST-SSR	(GA)12	136	Martis et al., 2013
2RS	*GRM0986*	TTTCCTCCCCATTAATCACCT	GATTGTTTGTTTGGGATGCAC	EST-SSR	(CTT)11	172	Martis et al., 2013
2RS	*GRM1243*	GGTGATGCTTCGATTTGTTTG	CCACTAATTCAAGTTGCCACA	EST-SSR	(GCCT)6	144	Martis et al., 2013
2RS	*REMS1203*	TTCGAAAGAGGATACCCAGC	GAGTCGATCACAAACGGGAT	EST-SSR	(GAA)5	131	Khlestkina et al., 2004
2RL	*REMS1238*	TACGTGGACGAGGAGGAGAC	TACCTACCATCACCACCCTG	EST-SSR	(CGG)5	250	Khlestkina et al., 2004
2RL	*REMS1251*	CAGCTTCATATGTTGCACGG	GTTCCCGATCTTGGATGAGA	EST-SSR	(CATA)5	225	Khlestkina et al., 2004
2RL	*REMS1208*	GAAAGTCGTCTCGACCCATC	GATGGCCACCATCATCTTCT	EST-SSR	(CGC)6	107	Khlestkina et al., 2004
2RL	*REMS1230*	GAGCAACAGCGACATCTTCA	ACCCAAGGCAAAAGGGTACT	EST-SSR	(AGC)6	267	Khlestkina et al., 2004
2RL	*SCM149*	GGATTGGATCTGAAGAAAGTC	CGATTCGCTTGAAAGTTTAC	EST-SSR	(TTTC)3	170	Hackauf and Wehling, 2002
2RL	*CINAU100*	ATCCAGTGGTCTGGAACAGG	ACAGAAGGGGCACAGCTAGA	STS		216	Zhuang et al., 2010
2RL	*CGG9*	CAGAGCAACAGCGACATCTTC	TCAACCCAAGGCAAAAGG	EST		200	Xu et al., 2012
2RL	*GRM0462*	GGCAGGCCCTGTAGCTATTA	ACGGCTACTAATGACATTTCC	EST-SSR	(TTGA)5	159	Martis et al., 2013
2RL	*GRM0079*	ACCACCATGGATGGACATCTT	CAAACAACAGTCGCCGTAGAT	EST-SSR	(TCT)14	136	Martis et al., 2013
2RL	*GRM1096*	TGCCTCATTAGCTATCGCAAC	ATGACGGGTAGGACTACATGC	EST-SSR	(AG)9	146	Martis et al., 2013
4RS	*REMS1160*	CTCGAGGAGGTTCGTTTCTG	ACCAGAGGAATCGCAAACAC	EST-SSR	(TAG)7	192	Khlestkina et al., 2004
4RS	*(TAG)_7_*	CTCGAGGAGGTTCGTTTCTG	ACCAGAGGAATCGCAAACAC	SSR		228	Nguyen et al., 2015
4RS	*KSUM62*	GGAGAGGATAGGCACAGGAC	GAGAGCAGAGGGAGCTATGG	EST		160	Xu et al., 2012
4RS	*GRM0554*	TTGCTTACTGCACATGGACCT	AGCGCTACAGATCGTCAACAT	EST-SSR	(GAT)6	145	Martis et al., 2013
4RS	*GRM0203*	CCCCTTCATCATCAAAGGATAA	ACATATGCCAGACACAATTCG	EST-SSR	(TC)8	156	Martis et al., 2013
4RS	*GRM0215*	TGGAGTTATGTCCAGTGCTCA	CACCTACGAACCGCATAGGTA	EST-SSR	(GC)9	123	Martis et al., 2013
4RL	*MAG1424*	TGAACATCAAGGGGCTGC	ACGACAGACATAAAGAAGAGCG	EST		260	Xu et al., 2012
4RL	*GRM0698*	GCTTCTTCTTCTTGCCCATCT	AGCAGCAGAGCATCTAACCAA	EST-SSR	(ACA)6	150	Martis et al., 2013
4RL	*GRM1178*	TTCTCCTCCCCAAGGTGTAGT	CCATCCATGATCCATCAATCT	EST-SSR	(AGC)6	143	Martis et al., 2013
4RL	*GRM0022*	CACTATACATCCGATCCCATCC	GTCACACTTCTGCTCGGAGAT	EST-SSR	(TCC)6	154	Martis et al., 2013

### Marker analysis of leaf rust at both stages and powdery mildew resistance at the adult-plant stage

At the adult-plant stage, we analyzed powdery mildew and leaf rust resistance when both diseases had thoroughly developed on Xuezao at around 50 dpi. Among 257 BC_2_F_1_ plants, 152 were found to be strongly resistant to both the wheat leaf rust isolate PHT and the powdery mildew isolate E09 (Figure S1D). Our marker analysis showed that all the resistant plants carried the 2R or 2RL chromosome, whereas plants possessing the 1R, 2RS, 3R, 4R, 5R, 6R, and 7R chromosomes appeared to be very susceptible to leaf rust. This indicates that 2RL from Sorento confers strong resistance to wheat powdery mildew and leaf rust diseases at the adult-plant stage (Figure S1D, Table 4). The result was consistent with that from BC_1_F_1_, where all plants resistant to leaf rust were also resistant to powdery mildew fungus. In contrast to the moderate resistance conferred by 2RL against powdery mildew at seedling stage, adult plants carrying this chromosome were strongly resistant (Figure S1D). Except for adult plants containing 4R and 2R, plants with other chromosomes of Sorento were moderately or highly susceptible against isolate E09.

**Table 4:** BC_2_F_1_ line sx-53 segregating for 2R and 4R chromosomes^a^.

**BC_2_F_1_ individuals**	**Reaction of powdery mildew at SS^b^**	**Reaction of powdery mildew at APS^c^**	**Reaction of leaf rust at APS^c^**	**4RS**		**4RL**		**2RS**		**2RL**	
				***REMS 1160***	***(TAG)_7_***	***MAG1424***	***GRM 0022***	***CGG62***	***SCM 153***	***SCM 149***	***REMS 1251***
sx-53-4	0	R	R	+	+	+	+	+	+	+	+
sx-53-20	0	R	R	+	+	+	+	+	+	+	+
sx-53-21	0	R	R	+	+	+	+	+	+	+	+
sx-53-8	1	R^d^	S	+	+	+	+				
sx-53-12	1	R	R	+	+	+	+	+	+	+	+
sx-53-27	1	R^d^	S	+	+	+	+				
sx-53-32	1	R	R	+	+	+	+	+	+	+	+
sx-53-24	1	R	R	+	+	+	+	+	+	+	+
sx-53-1	1	R^d^	S	+	+	+	+				
sx-53-6	2	R	R					+	+	+	+
sx-53-23	2	R	R					+	+	+	+
sx-53-25	2	R	R					+	+	+	+
sx-53-9	2	R	R					+	+	+	+
sx-53-10	2	R	R					+	+	+	+
sx-53-18	3	S	S								
sx-53-3	4	S	S								
sx-53-7	4	S	S								
sx-53-13	4	S	S								
sx-53-14	4	S	S								
sx-53-17	4	S	S								
sx-53-31	4	S	S								
sx-53-2	4	S	S								
sx-53-5	4	S	S								
sx-53-11	4	S	S								
sx-53-15	4	S	S								

a *Only one BC_2_F_1_ line derived from a BC_1_F_1_ plant (sx-53) was shown*.

b *SS, seedling stage*.

c *APS, adult-plant stage*.

d *Since plants carrying the 4R chromosome were not incompatible with leaf rust disease, the occurrence of powdery mildew uredospores was obscured by the leaf rust pustules; however, the resistance of these plants against powdery mildew was confirmed in the greenhouse at the adult-plant stage*.

Plants carrying the 4R chromosome of Sorento were strongly sensitive to leaf rust, resulting in our inability to distinguish the reaction to powdery mildew at the adult-plant stage in the field. Because the 4RL chromosome conferred high resistance to powdery mildew at the seedling stage, it was necessary to exclude the influence of leaf rust uredinia on the occurrence of powdery mildew at the adult-plant stage. Therefore, the powdery mildew resistance of BC_2_F_2_ plants carrying 4RL at the adult-plant stage was investigated in the greenhouse. As expected, plants carrying the 4RL of Sorento chromosome were completely incompatible with powdery mildew, which is consistent with their performance at the seedling stage (Figures S1C,E).

To test the seedling resistance against leaf rust, 50 BC_2_F_2_ plants derived from 5 BC_2_F_1_ carrying chromosome 2R and one plant carrying chromosome arm 2RL and showing resistance to leaf rust at the adult-plant stage were evaluated in the growth chamber. Eighteen and three plants containing chromosomes 2R and 2RL, respectively, were highly resistant to leaf rust (Figure S1B). The response of both seedling and adult plants carrying the 2R or 2RL chromosome to leaf rust was characterized by a strong hypersensitive reaction (Figures S1B,D). In conclusion, the 2RL chromosome transferred from Sorento confers resistance to leaf rust and powdery mildew, whereas the 4RL chromosome from Sorento contributes resistance only to wheat powdery mildew.

### Validation of 2R and 4R chromosomes by cytological analysis

**Figure 4:**
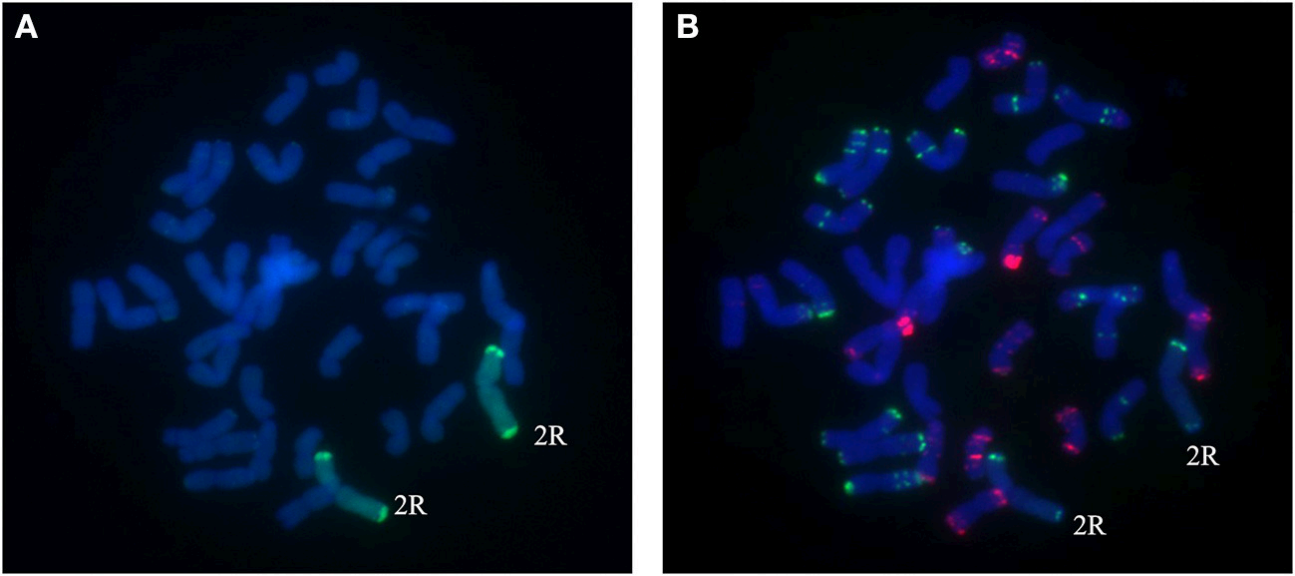
Chromosome composition of BC_2_F_3_ line 1289 by FISH **(B)** and GISH **(A)** using probe pAs1 (red) and probe pSc119.2 (green). A pair of 2R chromosomes from Sorento have been introduced into wheat.

**Figure 5:**
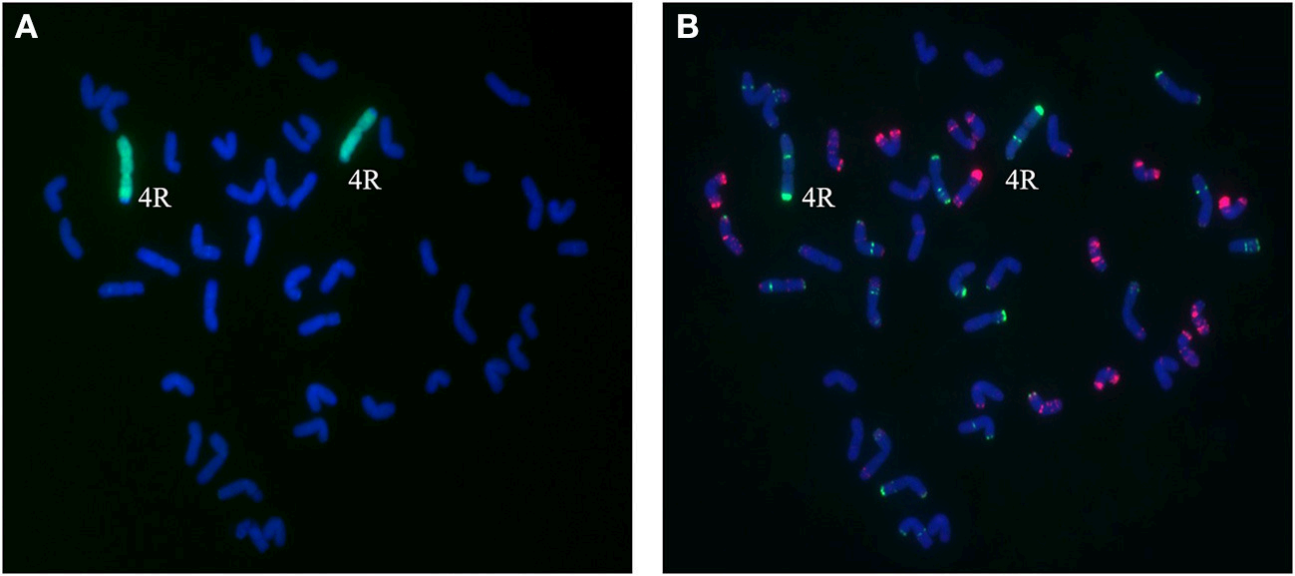
Chromosome composition of BC_2_F_3_ line 1419 by FISH **(B)** and GISH **(A)** using probe pAs1 (red) and probe pSc119.2 (green). Line 1,419 contains a pair of 4R chromosomes from Sorento.

**Figure 6:**
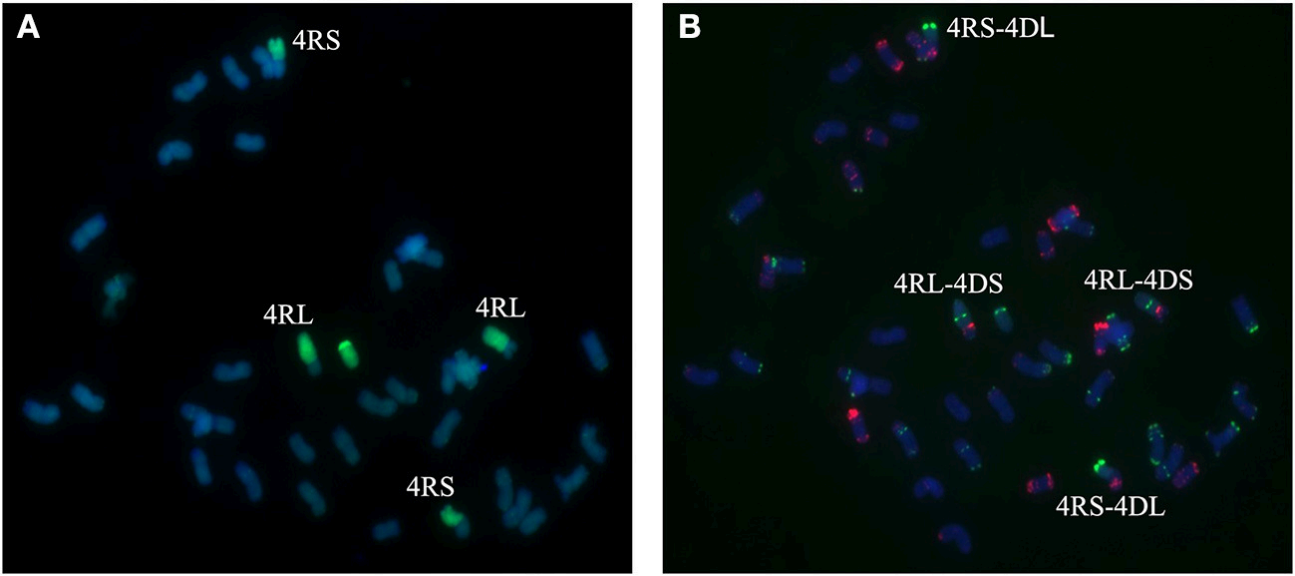
Chromosome composition of BC_2_F_3_ line 1204 by FISH **(B)** and GISH **(A)** using probe pAs1 (red) and probe pSc119.2 (green). Line 1,204 shows to be a 4R-4D reciprocal translocation in which the short arm of 4R chromosome was translocated to the long arm of 4D chromosome and the long arm of 4R chromosome was translocated to the short arm of 4D chromosome.

**Figure 7:**
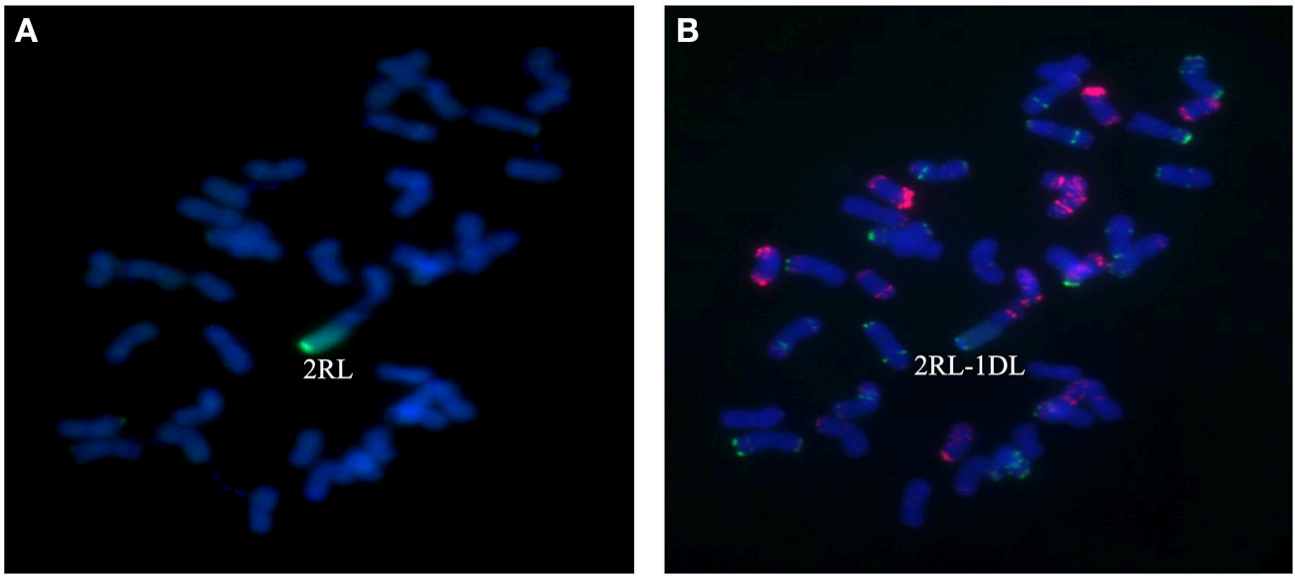
Chromosome composition of BC_2_F_3_ line 1,887 by FISH **(B)** and GISH **(A)** using probe pAs1 (red) and probe pSc119.2 (green). Line 1,887 was shown to be a monosomic 2RL-1DL translocation line in which the long arm of 2R was translocated to the long arm of 1D chromosome.

A total of 25 BC_2_F_3_ lines derived from BC_2_F_2_ plants which had been shown by marker analysis to contain either the 2R or the 4R chromosome were verified by GISH and FISH analysis. After inoculating seedlings of 25 BC_2_F_3_ lines with wheat *Bgt* isolate E09 and *Pt* isolate PHT, segregant and non-segregant lines for resistance against powdery mildew and leaf rust were identified. By cytological analysis, all plants contain 42 chromosomes. Among those, seven lines homozygous for leaf rust and powdery mildew resistance were presented to carry a pair of 2R chromosomes (Figures 4A,B), and six lines homozygous for powdery mildew resistance were shown to contain a pair of 4R chromosomes (Figures 5A,B). One line, 1,204, appears to contain a 4R-4D reciprocal translocation in which the arms 4RS and 4DL, or 4RL and 4DS, are interchanged (Figures 6A,B). Line 1,887 was shown to be a monosomic 2RL-1DL translocation line in which 2RLand 1DL were interchanged (Figures 7A,B). The rest of the BC_2_F_3_ lines segregating for leaf rust and powdery mildew resistance were determined to be monosomic substitution lines. In these lines, 2R chromosomes were substituted by a pair of 2D chromosomes of wheat for all BC_2_F_3_ lines homozygous for 2R, which was confirmed by the absence of PCR products of wheat 2D-derived SSR markers. However, the wheat chromosomes substituted by other rye chromosomes still remained unknown and need to be identified later. These results were consistent with the marker analysis and the newly developed lines have been applied in wheat breeding.

## Discussion

Triticale cultivar Sorento, which combines the superior stress tolerance of rye and high yield of *T. turgidum*, integrates the resistance advantages of different triticale lines. Crosses between hexaploid triticale and wheat are easier than those between rye and wheat due to better seed set (Hills et al., 2007), and because colchicine treatment and tedious embryo rescue are not required. The hexaploid feature also assists in the hybridization of triticale and wheat. Triticale cv. Sorento displayed outstanding resistance to wheat powdery mildew and leaf rust diseases as well as triticale diseases. However, the resistance has not been investigated and exploited in wheat breeding. Here, we successfully introduced resistance to wheat powdery mildew and leaf rust derived from hexaploid triticale cv. Sorento into susceptible wheat line Xuezao by backcrossing. The resulting BC_1_F_1_, BC_2_F_1_, BC_2_F_2_, and BC_3_F_1_ plants were genotyped with rye chromosome-specific markers and evaluated for resistance against powdery mildew and leaf rust at the seedling and adult-plant stages. We proved that the long arms of the 2R and 4R chromosomes from Sorento carry resistance genes against wheat powdery mildew and leaf rust diseases. The long arm of 4R confers high resistance to wheat powdery mildew at the seedling and adult-plant stages, and the long arm of 2R confers moderate powdery mildew resistance at the seedling stage and strong resistance at the adult-plant stage. Additionally, 2RL confers strong resistance toward wheat leaf rust accompanied by a strong hypersensitive reaction (HR). HR is a notable feature of race-specific response of R genes and is characterized by localized cell death supposed to avoid spread of biotroph pathogens to the healthy tissue of plants (Jones and Dangl, 2006). R genes mainly function throughout whole stage divergent from the adult-plant resistance (APR) which mainly takes place at adult stage and is characterized as non-race-specific (Periyannan et al., 2017). However, race-specific resistance and HR were also reported for APR genes such as *Lr12, Lr22*, and *Lr35* (Ellis et al., 2014), and the characteristics of the resistance of 2RL need to be determined systematically.

Fertility seems to be the main barrier in introducing resistance genes into bread wheat. In our crossing process, most of the early generations such as F_1_, BC_1_F_1_, and BC_2_F_1_ displayed inferior crossability with Xuezao and lower self-fertility rate. This may be due to the influences of maternal genotype among plants and meiotic disturbances (Oettler, 2005). The transmission rate of rye chromosomes in the F_1_ × Xuezao backcross is consistent with that of Lukaszewski et al. (1982). The lower transmission frequency of chromosome 3R and 6R appeared also in the BC_2_F_1_ backcross. By contrast, disomic substitutions for chromosomes 2R and 4R showed a high seed setting rate above 90%.

The powdery mildew resistance gene *Pm7* located on 2RLin the form of T4BS.4BL-2RL from Transec has been overcome and shows no resistance to E09 (Friebe et al., 1996; Zhuang, 2003; Zhang et al., 2010). Chromosome arm 2RL also carries the leaf rust resistance gene *Lr25*, however it has not contributed to wheat breeding improvement because the Transec translocation has an additional intercalary segment derived from 5RL (Friebe et al., 1996).

Hysing et al. (2007) reported that SLU translocation lines carrying T2BS.2RL were completely resistant to 17 powdery mildew isolates and 14 leaf rust isolates at the seedling stage and to the same mixture of powdery mildew isolates at the adult plant stage, however, adult-stage resistance conferred by T2BS.2RL against leaf rust was not well-described. The Xiaoyan 6-German White 2R (2D) chromosome substitution lines developed using the nullisomic back-cross procedure were also reported to confer resistance to powdery mildew isolates prevalent in northern China (An et al., 2006). However, the primer pair R1 R2 used for detecting 2R of German White did not amplify the 473 bp product in our resistant lines. Zhuang et al. (2010) demonstrated that the resistance to powdery mildew in H-J DA2RDS1R(1D) was provided by the 2RL from rye cv. Jingzhouheimai. However, markers *Xscm32, Xscm33, Xscm75*, and *Xcinau514* mapped on 2R from Jingzhouheimai amplified no products in Sorento or our resistant progeny. In light of its diverse genetic background and specificity of markers and the unique resistance phenotype of powdery mildew and leaf rust, we infer that the resistance loci on 2RL in Sorento may be novel.

Resistance derived from 4RL has been reported. Fu et al. (2014) showed that monotelosomic or ditelosomic addition lines of the long arm of rye chromosome 4R (4RL) from Kustro displayed immunity to mixed wheat powdery mildew (*Bgt*) isolates collected from Sichuan, China. The result was confirmed with 4RL-specific marker *SCIM808*_986_. To distinguish this segment, we used the same marker to analyze the resistance contributed by 4RL in Sorento and its progenies. Unexpectedly, no product was amplified. An et al. (2013) reported that Xiaoyan 6-German White 4R chromosome translocation line WR41-1 (T4BL.4RL and T7AS.4RS) showed high levels of resistance to powdery mildew. Markers *KSUM62* (4RS) and *MAG1424* (4RL) associated with powdery mildew resistance of WR41-1 also co-segregated with the resistance of 4R in our plants. However, whether the wheat powdery mildew resistance we identified represents a novel resistance locus on 4RL remains unclear.

Recombination between alien chromosomes and wheat chromosomes are limited and lots of efforts have been made to facilitate recombination between wheat and rye to exclude the disadvantageous chromatin. (Lukaszewski, 2000; Mago et al., 2002; Qi et al., 2007). Lack of recombination hampers the delimitation of resistance genes on a block of rye chromosome arm where multiple resistance genes may be responsible for a particular resistance such as the wheat-*Haynaldia villosa* translocation line T6VS.6AL (Cao et al., 2011; He et al., 2017). Although both 2RL and 4RL from Sorento confer resistance at seedling and adult stage, it is not clear whether the resistance at both stages was controlled by the same gene or a cluster of genes. Genetic and cytogenetic methods need to be put into practice to promote recombination and minimize the adverse effects brought by the rye chromatin.

## Author contributions

FL and CX: designed the research; FL, YL, LC, PL, MG, QZ, and LQ: did the cross work and phenotype evaluation; FL and LC: completed the molecular marker and cytological analysis; CX and QS: provided the material; FL: wrote the paper.

### Conflict of interest statement

The authors declare that the research was conducted in the absence of any commercial or financial relationships that could be construed as a potential conflict of interest.
