# Supplementary material for: Simultaneous Transfer of Leaf Rust and Powdery Mildew Resistance Genes from Hexaploid Triticale Cultivar Sorento into Bread Wheat
Source: Front Plant Sci. 2018 Feb 5;9:85. doi: 10.3389/fpls.2018.00085 (PMC5807375; doi:10.3389/fpls.2018.00085)
Supplement: Supplementary file 1 [file Table1.PDF]

Simultaneous Transfer of Leaf Rust and Powdery Mildew Resistance Genes From Hexaploid Triticale cultivar Sorento into Bread Wheat

Feng Li,Yinghui Li, Lirong Cao, Peiyuan Liu, Miaomiao Geng, Qiang Zhang, Lina Qiu,Qixin Sun,Chaojie Xie*
Key Laboratory of Crop Heterosis and Utilization, Ministry of Education, State Key Laboratory of Agro-biotechnology, Beijing Key Laboratory of Crop Genetic Improvement,
China Agricultural University, Beijing, 100193, China
* Corresponding author:
Chaojie Xie: xiecj127@126.com

Table S1 All rye markers used for identification of seven rye chromosomes

No.	Chromosome	Marker	Forward sequence	Reverse sequence	Type	SSR	Expected size(bp)	EST accession no.	Reference	Characteristics
1	1R	*REMS1303*	TAGCACCACTCGCTCTCTCA	TTTCCCATCAGAAAAATCGC	EST-SSR	(CTC)5	273	BF146157	Khlestkina et al. 2004	dominant[d]
2	1R	*REMS1280*	CAACGGCATGGAGTACCCT	GAAGTTAACCTGCGGGAACA	EST-SSR	(CCT)6	163	BF145382	Khlestkina et al. 2004	dominant
3	2R	*REMS1238*	TACGTGGACGAGGAGGAGAC	TACCTACCATCACCACCCTG	EST-SSR	(CGG)5	250	BE637241	Khlestkina et al. 2004	co-dominant[e]
4	2R	*REMS1251*	CAGCTTCATATGTTGCACGG	GTTCCCGATCTTGGATGAGA	EST-SSR	(CATA)5	225	BE704539	Khlestkina et al. 2004	dominant
5	2R	*REMS1203*	TTCGAAAGAGGATACCCAGC	GAGTCGATCACAAACGGGAT	EST-SSR	(GAA)5	131	BE586786	Khlestkina et al. 2004	dominant
6	2R	*REMS1230*	GAGCAACAGCGACATCTTCA	ACCCAAGGCAAAAGGGTACT	EST-SSR	(AGC)6	267	BE588133	Khlestkina et al. 2004	co-dominant
7	3R	*REMS1254*	AAAATACGGAGGAGGCAGGT	ACATCAACAAGATCGTGGGC	EST-SSR	(CGG)5	275	BE704639	Khlestkina et al. 2004	dominant
8	3R	*REMS1323*	TTTGTTGCTAAGCTCCCTTCA	GGTGATCCATGAACAAACCC	EST-SSR	(CT)7atctatcta(TC)7	292	BE704639	Khlestkina et al. 2004	dominant
9	4R	*REMS1160*	CTCGAGGAGGTTCGTTTCTG	ACCAGAGGAATCGCAAACAC	EST-SSR	(TAG)7	192	BE494651	Khlestkina et al. 2004	dominant
10	5R	*REMS1264*	AAAACCATCCACACATCCGT	GAACTCGCTCTTCATCCTCG	EST-SSR	(CGTC)5	246	BE705252	Khlestkina et al. 2004	dominant
11	5R	*REMS1266*	AAAGGAAAACCACCTCAGGG	GCATTTTGCAGGAGAAGCAT	EST-SSR	(GA)8	166	BE705296	Khlestkina et al. 2004	dominant
12	5R	*REMS1237*	GCAATCTCAGATCCTACGGC	GCTTCTGACTGAGCGAACCT	EST-SSR	(TAGC)5	252	BE637153	Khlestkina et al. 2004	dominant
13	5R	*REMS1186*	CGTCTCGTCGCGTAAAAACT	ACCTACCCACCCACCGAT	EST-SSR	(CAC)5	185	BE495963	Khlestkina et al. 2004	ND[b]
14	5R	*REMS1205*	TTGTTTTGCCAAAGAAGGCT	TCACATCATGGAGGAACCAA	EST-SSR	(ACAT)6	245	BE586813	Khlestkina et al. 2004	dominant
15	6R	*REMS1152*	AGATCAGATCAGAGCCGGAG	GCGTCCACGAGTAGTTGTCA	EST-SSR	(GCA)5	215	BE494415	Khlestkina et al. 2004	co-dominant
16	6R	*REMS1247*	CAGCAGCACACACTCGGTAT	AATGGCGCATTAAGCAGTTT	EST-SSR	(TTC)7	276	BE704499	Khlestkina et al. 2004	dominant
17	7R	*REMS1188*	CGGGTATCACATGTCGTCAG	TCAGCGCAAAACACTACTGC	EST-SSR	(TC)7	135	BE496047	Khlestkina et al. 2004	dominant
18	7R	*REMS1234*	TTTACTAAACCAAACCAGCGG	CGTTCTCGATCCGCTTTATC	EST-SSR	(AGC)6	220	BE637039	Khlestkina et al. 2004	dominant
19	7R	*REMS1187*	ACAGGAGGTTTCAGCAGCAT	CACGTGTTGTTTCCCTTCCT	EST-SSR	(CAA)5	179	BE496005	Khlestkina et al. 2004	dominant
20	7R	*REMS1162*	ATGACCTGGTGAGGTTCGTC	CGTCTTGAAAGGCTCGTTGT	EST-SSR	(GCC)5	164	BE494705	Khlestkina et al. 2004	dominant
21	7R	*REMS1281*	TACTGGTCGTACAGGGCCTC	CATCCATTTCCCTGCAAGAT	EST-SSR	(GAA)5	279	BF145397	Khlestkina et al. 2004	co-dominant
22	7R	*REMS1253*	TCCCACATATGTCCAGACGA	AGAGGAATACTCGGATGGGG	EST-SSR	(ATAG)5	247	BE704638	Khlestkina et al. 2004	ND
23	1R	*SCM39*	GACCTCAGTGGAGCCTCTAGGT	GGACATCTGCCGTGACAATACC	SSR	(GT)8(GC)6… (GT)53	230		Saal and Wricke 1999	dominant
24	1RS	*SCM9*	TGACAACCCCCTTTCCCTCGT	TCATCGACGCTAAGGAGGACCC	SSR	(GT)8	220		Saal and Wricke 1999	dominant
25	1RL	*SWES1119*	GAAACCCACCTCCCTTAC	AGAGCCTACCATCCCATC	EST		400	CJ653076	Xu et al. 2012	dominant
26	1RL	*SWES1128*	GCCTACCATCCCATCTTC	CCTCCCTTACCCTATCAA	EST		300	CJ545184	Xu et al. 2012	dominant
27	3R	*3R_(GA)6*	AAAATACGGAGGAGGCAGGT	ACATCAACAAGATCGTGGGC	SSR		252		Nguyen et al.2015	dominant
28	3RS	*SCM102*	AACAAGTGCAGAACTCGCGT	CAGAAAGTCTTGGGGCCAG	SSR	(AG)27	208		Saal and Wricke 1999	dominant
29	4RS	$(TAG)_{7}$	CTCGAGGAGGTTCGTTTCTG	ACCAGAGGAATCGCAAACAC	SSR		228		Nguyen et al.2015	dominant
30	4RS	*KSUM62*	GGAGAGGATAGGCACAGGAC	GAGAGCAGAGGGAGCTATGG	EST		160	BE585783	Xu et al. 2012	dominant
31	4RL	*MAG1424*	TGAACATCAAGGGGCTGC	ACGACAGACATAAAGAAGAGCG	EST		260	TC265631	Xu et al. 2012	dominant
32	4RL	*CGG49*	GAACGCAAGCACTTCTCA	GCTCCTTTCTAAGCCTGTC	EST		1000	BE586668	Xu et al. 2012	NA[a]
33	4RL	*SCIM808*$_{986}$	CACGAGCCGATCCCACTAT	ATGGACCCGTACGTCTACAAA					Fu et al.2014	NP[c]
34	6RL	*SWES78*	GGAAGGCAACAACAAAGTG	CAGGCTGTGGACGGAGAT	EST		230	CK204959	Xu et al. 2012	co-dominant
35	6RL	*SWES206*	TGCCATCGACAACTACCAA	CGTTTCTCCTCCAATCCAG	EST		180	CD898587	Xu et al. 2012	co-dominant
36	6RL	*SWES231*	AAGCATCCTACATAGCCCTCA	GGAGACCACTCGGCAGAAA	EST		260	CD916038	Xu et al. 2012	dominant
37	6RL	*DUPW111*	CTTATCGCTGTTCATCGTCG	GGAAGGGAAAGTACAACTCGC	EST		150	No data	Xu et al. 2012	co-dominant
38	2R	*R1*	CCCAGCAACAACAACCGTCGATTC	AATCGACGGTTGTTGTTGCTGGG					An et al.2006	NA
39	2RS	*CGG62*	GCCCTCGACGACATGAAA	CGCTTGCCGGTCTTGTAT	EST		290	BE587051	Xu et al. 2012	co-dominant
40	2RS	*SCM153*	CACTATGATAACCGTACCTCAA	AATACTGCACGTAGGAATCAAC	EST-SSR	(AT)9	170	BE705420	Hackauf and Wehling 2002	dominant
41	2RS	*XCINAU174*	GCTTTAGTATGGGTGCTGC	CATCTCCTCTGGAACGGTAT	STS			TC251256	Zhuang et al. 2010	NP
42	2RS	*SCM32*	GCTACACTGATGTATTCAACGA	GTCGTCGTCATCGTCATC	EST-SSR	(ACG)7*(ATGACG)3*(ACG)2	130	BE494297	Hackauf and Wehling 2002	NA
43	2RL	*SCM33*	TGGCACTGCAACTATCGTACAC	TCGTCGTCGTCGTCGTCAT	EST-SSR	(ACG)15 imp.	176	BE494611	Hackauf and Wehling 2002	NA
44	2RL	*SCM38*	ACCTCATCTCATCTTCGTCTT	ATGTCGATTCCAGATCCTC	EST-SSR	(AGC)6	192	BE587446	Hackauf and Wehling 2002	NP
45	2RL	*SCM71*	AAGCTGAGGGAAAGATGAG	GATCCTCACCAGCTGAATC	EST-SSR	(CTC)6	159	BE587485	Hackauf and Wehling 2002	NP
46	2RL	*SCM75*	TTTTCTATCTCAGCGATTCATGC	TCCTGAGATCAAGTGCGTGTG	SSR	(CA)7(CT)15… (CA)10	191		Saal and Wricke 1999	NP
47	2RL	*SCM149*	GGATTGGATCTGAAGAAAGTC	CGATTCGCTTGAAAGTTTAC	EST-SSR	(TTTC)3	170	BE586531	Hackauf and Wehling 2002	co-dominant
48	2RL	*XCINAU100*	ATCCAGTGGTCTGGAACAGG	ACAGAAGGGGCACAGCTAGA	STS		216	BE705070	Zhuang et al. 2010	dominant
49	2RL	*XCINAU514*	AACACCACGGAGGAGGAGC	TGACGGTGAGGACGCACTCG	STS		247	CK202473	Zhuang et al. 2010	NA
50	2RL	*SWES120*	CGACGACTACCTCCTCAAGAA	GAACAGGCAACGACGACAG	EST		120	AL811749	Xu et al. 2012	dominant
51	2RL	*CGG8*	ATCCATCCCATCCCGTCTC	TGGCTACACTCGCTCTCGTC	EST		90	BE586891	Xu et al. 2012	ND
52	2RL	*CGG9*	CAGAGCAACAGCGACATCTTC	TCAACCCAAGGCAAAAGG	EST		200	BE588133	Xu et al. 2012	co-dominant
53	2RS	*GRM1082*	TCTAGCTCATCAAGTGCTTACCA	GTTCCGCTGAGATGAACCATA	EST-SSR	(GA)12	136		Martis et al.2013	dominant
54	2RS	*GRM0986*	TTTCCTCCCCATTAATCACCT	GATTGTTTGTTTGGGATGCAC	EST-SSR	(CTT)11	172		Martis et al.2013	dominant
55	2RS	*GRM1243*	GGTGATGCTTCGATTTGTTTG	CCACTAATTCAAGTTGCCACA	EST-SSR	(GCCT)6	144		Martis et al.2013	co-dominant
56	2RL	*GRM0462*	GGCAGGCCCTGTAGCTATTA	ACGGCTACTAATGACATTTCC	EST-SSR	(TTGA)5	159		Martis et al.2013	dominant
57	2RL	*GRM0079*	ACCACCATGGATGGACATCTT	CAAACAACAGTCGCCGTAGAT	EST-SSR	(TCT)14	136		Martis et al.2013	dominant
58	2RL	*GRM1096*	TGCCTCATTAGCTATCGCAAC	ATGACGGGTAGGACTACATGC	EST-SSR	(AG)9	146		Martis et al.2013	dominant
59	4RS	*GRM0554*	TTGCTTACTGCACATGGACCT	AGCGCTACAGATCGTCAACAT	EST-SSR	(GAT)6	145		Martis et al.2013	dominant
60	4RS	*GRM0203*	CCCCTTCATCATCAAAGGATAA	ACATATGCCAGACACAATTCG	EST-SSR	(TC)8	156		Martis et al.2013	dominant
61	4RS	*GRM0215*	TGGAGTTATGTCCAGTGCTCA	CACCTACGAACCGCATAGGTA	EST-SSR	(GC)9	123		Martis et al.2013	co-dominant
62	4RL	*GRM0698*	GCTTCTTCTTCTTGCCCATCT	AGCAGCAGAGCATCTAACCAA	EST-SSR	(ACA)6	150		Martis et al.2013	dominant
63	4RL	*GRM1178*	TTCTCCTCCCCAAGGTGTAGT	CCATCCATGATCCATCAATCT	EST-SSR	(AGC)6	143		Martis et al.2013	co-dominant
64	4RL	*GRM0022*	CACTATACATCCGATCCCATCC	GTCACACTTCTGCTCGGAGAT	EST-SSR	(TCC)6	154		Martis et al.2013	dominant

[a]NA (no amplification) means the markers amplify no products in Sorento and Xuezao.

[b]ND (no difference) means the markers show no difference between Sorento and Xuezao.

[c]NP (no polymorphism) signifies the markers are not specific for a rye chromosome.

[d]"Dominant" means polymorphic markers specific for a rye chromosome only amplify products in one of the parents.

[e]"Co-dominant" means polymorphic markers specific for a rye chromosome amplify different products in both parents.
