# Supplementary figures and images for: Simultaneous Transfer of Leaf Rust and Powdery Mildew Resistance Genes from Hexaploid Triticale Cultivar Sorento into Bread Wheat
Source: Front Plant Sci. 2018 Feb 5;9:85. doi: 10.3389/fpls.2018.00085 (PMC5807375; doi:10.3389/fpls.2018.00085)

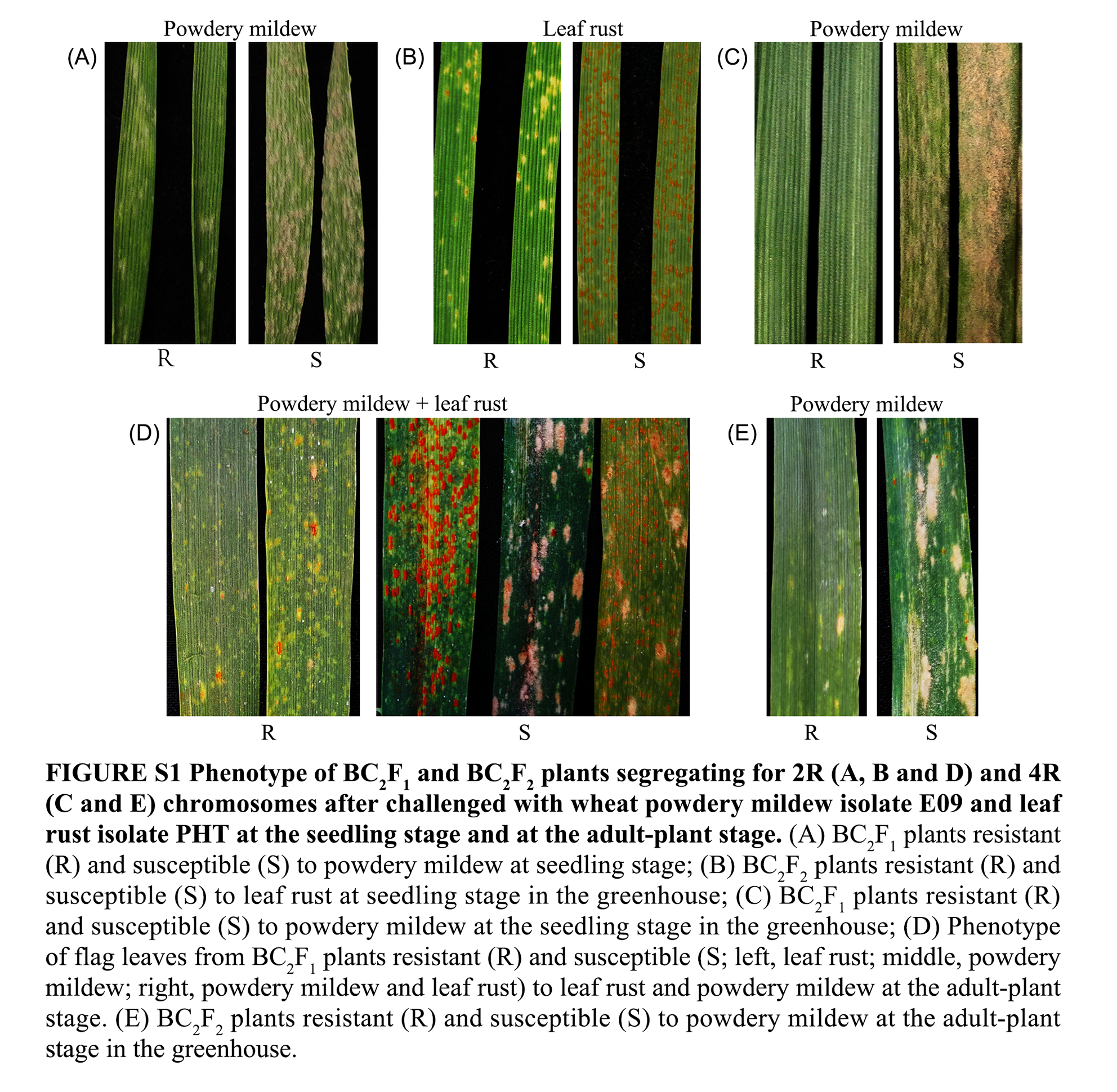

Supplement: Supplementary file 2 [file Image1.TIF]
